# A retrospective analysis of risk factors for massive hemoptysis in patients with bronchiectasis

**DOI:** 10.1186/s12890-022-02006-x

**Published:** 2022-06-02

**Authors:** Ling Luo, Jing Luo, Yu Jiang

**Affiliations:** Department of Respiratory and Critical Medicine, University-Town Hospital of Chongqing Medical University, Chongqing, 401331 China

**Keywords:** Bronchiectasis, Massive hemoptysis, Risk factors

## Abstract

**Background:**

Massive hemoptysis is a common and fatal complication of bronchiectasis. However, the risk factors for massive hemoptysis in patients with bronchiectasis have not yet been reported. This study investigated the potential risk factors for massive hemoptysis in patients with bronchiectasis.

**Methods:**

This retrospective study included patients with bronchiectasis and their data were obtained from medical records. The risk factors for massive hemoptysis were evaluated by multivariate analysis of patient characteristics, medical history, and computed tomography imaging data, including the number of lesions, lesion location, and laboratory findings.

**Results:**

Among 379 patients, 61 (16.09%) experienced severe hemoptysis. Multivariate analysis revealed that diabetes (odds ratio (OR) 2.885; 95% confidence interval (CI) 1.009–8.247), lesions involving two lobes (OR 4.347; 95% CI 1.960–9.638) and three lobes (OR 2.787; 95% CI 1.055–7.363) were significant predictors of severe hemoptysis. However, a disease course between 1 and 5 years (OR 0.300; 95% CI 0.112–0.801) and involvement of the left lower lobe (OR 0.394; 95% CI 0.196–0.793) were protective factors for the prevention of massive hemoptysis. Lesions in the right upper lobe were more likely to cause massive hemoptysis (OR 1.458) than involvement of other lobes.

**Conclusions:**

Diabetes and lesions involving two and three lobes, were risk factors for massive hemoptysis in patients with bronchiectasis. Disease duration between 1 and 5 years and involvement of the left lower lobe were protective factors, while lesions in the right upper lobe had a stronger relationship with massive hemoptysis in patients with bronchiectasis.

## Introduction

Hemoptysis is a daily diagnostic challenge and is associated with potentially life-threatening medical conditions [1, 2]. Massive hemoptysis is a life threatening condition with high mortality rates [3]. Bronchiectasis is one of the most common causes of massive hemoptysis [4, 5] and is also a frequent cause of hospitalization [6]. Mondoni M reported that bronchiectasis is the leading predictor of hemoptysis relapse [7]. As a result of the significant negative impact of severe hemoptysis in patients with bronchiectasis, it is certainly important for us to understand its risk factors and prevent patients from developing massive hemoptysis. The risk factors for severe hemoptysis in patients with bronchiectasis are not well known. Therefore, this study aimed to investigate the potential risk factors associated with massive hemoptysis in patients with bronchiectasis.

## Materials and methods

### Study population

We retrospectively analyzed the medical records of patients with bronchiectasis from the University Town Hospital of Chongqing Medical University in Chongqing, China. The cases included were consecutive patients with bronchiectasis diagnosed by typical chest computed tomography (CT) scan after admission between January 2014 and June 2021. The features of bronchiectasis on a typical chest computed tomography (CT) scan include abnormally widened and thickened airways that have an irregular wall, lack of tapering, and/or visibility of the airway in the periphery of the lung [8]. The data on the characteristics and clinical status of the patients on the first day of admission were collected. The study was approved by the Institutional Review Board of University-town Hopspital of Chongqing Medical University and the requirement for informed consent was waived because of the retrospective nature of the study.

### Potential risk factors

Potential variables include the following patient characteristics at hospital admission: clinical course, history of bronchial artery embolism, previous history of massive hemoptysis, demographic variables, medical history, imaging features, and laboratory findings. Demographic variables collected for the study included age (< 40 years, ≥ 40 and < 50 years, ≥ 50 and < 60 years, ≥ 60 and < 70 years, and ≥ 70 years), sex, and smoking status (never, current, and former). Medical history including the disease course (≤ 1 year, > 1 and ≤ 5 years, > 5 and ≤ 10 years, and > 10 years), presence of comorbidities (chronic obstructive pulmonary disease, diabetes, hypertension, pulmonary fungus, and pneumonia), and the use of aspirin and anticoagulation, were obtained. The imaging features collected included the locations of abnormalities on chest computed tomography (CT) imaging. Laboratory findings including platelet counts and D-dimer levels were obtained.

### Massive hemoptysis definitions

Massive hemoptysis was defined based on the Chinese Expert Recommendation for Diagnosis and Treatment of Massive Hemoptysis, given the extensive acceptance of this guideline [9]. Massive hemoptysis is defined as blood volume > 500 ml within 24 h, > 100 ml within one hour, or > 100 ml in each episode of hemoptysis. Moreover, any life-threatening hemoptysis and any hemoptysis that may cause airway obstruction and asphyxia were also considered as massive hemoptysis [10, 11]. The definition of massive hemoptysis is consistent with some definitions reported in the international literature [5, 12].

### Statistical methods

The data were double-checked before entry into the computer. Mean differences between the two groups were evaluated by using univariate analysis (Pearson’s chi-squared test, calibration chi-squared test, and Fisher’s exact test for categorical variables). Multivariate logistic regression analysis based on AIC regulations was performed for factors found to have a *P* value < 0.05 in the univariate analysis, to identify the risk factors for massive hemoptysis in patients with bronchiectasis. The risk factors are presented as odds ratios (ORs) with a 95% confidence interval (CI). Subsequently, the independent factors were taken as control variables, and five different lobes were included to observe their different risks for massive hemoptysis. Statistical analyses were performed using SPSS version 26.0, and statistical significance was set at *P* < 0.05.

## Results

Of the 379 patients with bronchiectasis, 61 had massive hemoptysis, of whom 40 (20.3%) were male. Most patients were between 60 and 70 years of age, but 18 (19.15%) patients who had massive hemoptysis were more than 70 years of age. Patients who were current smokers had a higher incidence (25%) of massive hemoptysis. A total of 124 (32.72%) patients were diagnosed with bronchiectasis within the previous year, of whom 27 (21.77%) had massive hemoptysis. Three patients received anticoagulation therapy, and one of them had severe hemoptysis. Three (33.3%) patients had invasive pulmonary aspergillosis comorbidities. Eighty-seven patients had pulmonary lesions in two lobes, among whom the incidence of massive hemoptysis among them was 27.59%.

In the univariate analysis, massive hemoptysis was more likely to occur in patients who had not stopped smoking (*P* = 0.028). It was less likely to occur in women (*P* = 0.022) and in patients with disease course between 1 and 5 years (*P* = 0.016). Massive hemoptysis was also more likely to occur when lesions involved two lobes (*P* = 0.004); however, patients with lesions located in the left lower lobes were less likely to develop massive hemoptysis (*P* = 0.03) (Table 1).

**Table 1:** Univariate analysis for the association between risk factors and massive hemoptysis in patients with bronchiectasis

	Massive hemoptysis			*P* (χ^2^)	OR (95% CI)	*P* (OR)
	Total (n = 379)	No (n = 318)	Yes (n = 61)			
Gender				0.020*		
Male	197 (51.98)	157 (79.70)	40 (20.30)		Ref	
Female	182 (48.02)	161 (88.46)	21 (11.54)		0.512 (0.289, 0.907)	0.022*
Age (years)				0.915		
< 40	23 (6.07)	19 (82.61)	4 (17.39)		Ref	
(40,50)	61 (16.09)	52 (85.25)	9 (14.75)		0.822 (0.226, 2.986)	0.766
(50,60)	93 (24.54)	79 (84.95)	14 (15.05)		0.842 (0.249, 2.848)	0.782
(60,70)	108 (28.50)	92 (85.19)	16 (14.81)		0.826 (0.248, 2.748)	0.755
≥ 70	94 (24.80)	76 (80.85)	18 (19.15)		1.125 (0.341, 3.714)	0.847
Smoking				0.073		
Never	277 (73.09)	238 (85.92)	39 (14.08)		Ref	
Current	72 (19.00)	54 (75.00)	18 (25.00)		2.034 (1.081, 3.826)	0.028*
Former	30 (7.92)	26 (86.67)	4 (13.33)		0.939 (0.311, 2.837)	0.911
Course (years)				0.075		
≤ 1	124 (32.72)	97 (78.23)	27 (21.77)		Ref	
(1,5]	74 (19.53)	68 (91.89)	6 (8.11)		0.317 (0.124, 0.809)	0.016*
(5,10]	74 (19.53)	61 (82.43)	13 (17.57)		0.766 (0.367, 1.597)	0.476
> 10	107 (28.23)	92 (85.98)	15 (14.02)		0.586 (0.293, 1.171)	0.130
PTMH				0.242		
No	357 (94.20)	302 (84.59)	55 (15.41)		Ref	
Yes	22 (5.80)	16 (72.73)	6 (27.27)		2.059 (0.772, 5.493)	0.149
Hypertension				0.334		
No	320 (84.43)	271 (84.69)	49 (15.31)		Ref	
Yes	59 (15.57)	47 (79.66)	12 (20.34)		1.412 (0.699, 2.853)	0.336
Diabetes				0.154		
No	359 (94.72)	304 (84.68)	55 (15.32)		Ref	
Yes	20 (5.28)	14 (70.00)	6 (30.00)		2.369 (0.873, 6.430)	0.091
COPD				0.658		
No	306 (80.74)	258 (84.31)	48 (15.69)		Ref	
Yes	73 (19.26)	60 (82.19)	13 (17.81)		1.165 (0.594, 2.285)	0.658
Asprin				1.000		
No	366 (96.57)	307 (83.88)	59 (16.12)		Ref	
Yes	13 (3.43)	11 (84.62)	2 (15.38)		0.946 (0.204, 4.378)	0.943
Anticoagulation				0.410		
No	376 (99.21)	316 (84.04)	60 (15.96)		Ref	
Yes	3 (0.79)	2 (66.67)	1 (33.33)		2.633 (0.235, 29.504)	0.432
Invasive pulmonary aspergillosis				0.334		
No	370 (97.63)	312 (84.32)	58 (15.68)		Ref	
Yes	9 (2.37)	6 (66.67)	3 (33.33)		2.690 (0.654, 11.060)	0.170
Pneumonia				0.658		
No	306 (80.74)	258 (84.31)	48 (15.69)		Ref	
Yes	73 (19.26)	60 (82.19)	13 (17.81)		1.165 (0.594, 2.285)	0.658
PLT (× 10^9^/L)				0.698		
Normal	317 (83.64)	268 (84.54)	49 (15.46)		Ref	
Low	44 (11.61)	35 (79.55)	9 (20.45)		1.406 (0.636, 3.109)	0.399
High	18 (4.75)	15 (83.33)	3 (16.67)		1.094 (0.305, 3.920)	0.890
D-dimer (mg/l)				0.620		
Normal	276 (72.82)	230 (83.33)	46 (16.67)		Ref	
High	103 (27.18)	88 (85.44)	15 (14.56)		0.852 (0.453, 1.604)	0.620
Lobes				0.012*		
1	128 (33.77)	113 (88.28)	15 (11.72)	a	Ref	
2	87 (22.96)	63 (72.41)	24 (27.59)	b	2.870 (1.404, 5.866)	0.004**
3	59 (15.57)	49 (83.05)	10 (16.95)	ab	1.537 (0.646, 3.661)	0.331
4	31 (8.18)	29 (93.55)	2 (6.45)	ab	0.520 (0.112, 2.401)	0.402
5	74 (19.53)	64 (86.49)	10 (13.51)	ab	1.177 (0.500, 2.773)	0.709
Left upper lobe				0.732		
No	185 (48.81)	154 (83.24)	31 (16.76)		Ref	
Yes	194 (51.19)	164 (84.54)	30 (15.46)		0.909 (0.525, 1.572)	0.732
Left lower lobe				0.029*		
No	128 (33.77)	100 (78.12)	28 (21.88)		Ref	
Yes	251 (66.23)	218 (86.85)	33 (13.15)		0.541 (0.310, 0.943)	0.030*
Right upper lobe				0.137		
No	237 (62.53)	204 (86.08)	33 (13.92)		Ref	
Yes	142 (37.47)	114 (80.28)	28 (19.72)		1.518 (0.873, 2.640)	0.139
Right middle lobe				0.595		
No	187 (49.34)	155 (82.89)	32 (17.11)		Ref	
Yes	192 (50.66)	163 (84.90)	29 (15.10)		0.862 (0.498, 1.491)	0.595
Right lower lobe				0.801		
No	192 (50.66)	162 (84.38)	30 (15.62)		Ref	
Yes	187 (49.34)	156 (83.42)	31 (16.58)		1.073 (0.620, 1.856)	0.801

*PTMH* previous times of massive hemoptysis, *COPD* chronic obstructive pulmonary disease, *PLT* platelet

*P < 0.05; **P < 0.01; ***P < 0.001; a,b: multiple comparisons were statistically significant; ab: multiple comparisons were not significant

Multivariate analysis revealed that the presence of diabetes (OR 2.885; 95% CI 1.009–8.247; *P* = 0.048), lesions in two lobes (OR 4.347; 95% CI 1.960–9.638; *P* < 0.001), and lesions in three lobes (OR 2.787; 95% CI 1.055–7.363; *P* = 0.039) were significantly associated with massive hemoptysis (Table 2). It also revealed that a disease course between 1 and 5 years (OR 0.300; 95% CI 0.112–0.801; *P* = 0.016) and involvement of the left lower lobe (OR 0.394; 95% CI 0.196–0.793; *P* = 0.009) were protective factors for the prevention of massive hemoptysis. We considered these risk factors as control variables and found that lesions in the right upper lobe were more likely to cause massive hemoptysis (OR 1.458), while lesions in the left lower lobe were less likely to cause massive hemoptysis (OR 0.550) (Table 3).

**Table 2:** Multivariate analysis to determine the influencing factors for massive hemoptysis among patients with bronchiectasis

Variable	OR	95% CI	*P* value
Gender			
Male	Ref		
Female	0.565	0.307–1.043	0.068
Course (years)			
≤ 1	Ref		
(1,5]	0.3000	0.112–0.801	0.016
(5,10]	0.850	0.382–1.890	0.690
> 10	0.784	0.357–1.724	0.545
Diabetes			
No	Ref		
Yes	2.885	1.009–8.247	0.048
Lobes			
1	Ref		
2	4.347	1.960–9.638	< 0.001
3	2.787	1.055–7.363	0.039
4	1.017	0.201–5.137	0.984
5	2.431	0.844–6.999	0.100
Left lower lobe			
No	Ref		
Yes	0.394	0.196–0.793	0.009

*CI* confidence interval, *Ref.* reference, *OR* odds ratio

**Table 3:** The relationship between the involvement of different pulmonary lobes and massive hemoptysis in patients with bronchiectasis

	Variable	Se	z	Wald	*P*	OR (95% CI)
Controlling factors	(Intercept)	0.326	− 3.140	9.858	0.002	
	Gender					
	Male					
	Female	0.308	− 1.958	3.833	0.050	0.547 (0.300, 1.001)
	Course (years)					
	≤ 1	0.494	− 2.064	4.262	0.039	0.361 (0.137, 0.950)
	(1,5]	0.408	− 0.586	0.343	0.558	0.787 (0.354, 1.751)
	(5,10]	0.400	− 0.844	0.712	0.399	0.713 (0.325, 1.563)
	> 10	0.494	− 2.064	4.262	0.039	0.361 (0.137, 0.950)
	Diabetes					
	No					
	Yes	0.531	1.840	3.387	0.066	2.655 (0.939, 7.512)
Independent variables	Left upper lobe					
	No					
	Yes	0.325	0.141	0.020	0.888	1.047 (0.554, 1.978)
	Left lower lobe					
	No					
	Yes	0.320	− 1.865	3.476	0.062	0.550 (0.294, 1.031)
	Right upper lobe					
	No					
	Yes	0.315	1.196	1.430	0.232	1.458 (0.786, 2.704)
	Right middle lobe					
	No					
	Yes	0.332	− 0.496	0.246	0.620	0.848 (0.443, 1.625)
	Right lower lobe					
	No					
	Yes	0.338	0.733	0.538	0.463	1.281 (0.661, 2.484)

## Discussion

In this retrospective study, we investigated the risk factors for massive hemoptysis in patients with bronchiectasis and found that the presence of diabetes, lesions in two lobes, and involvement of the right upper lobe were associated with severe hemoptysis. However, this study also revealed that shorter disease courses (between 1 and 5 years) and patients with lesions involving the left lower lobe were at a decreased risk of developing massive hemoptysis.

The most common causes of massive hemoptysis include bronchiectasis, tuberculosis, lung cancer, necrotizing pneumonia, and cryptogenic hemoptysis [11, 13]. A multicenter study demonstrated that 2.22% of patients with bronchiectasis developed massive hemoptysis [5]. In the present study, massive hemoptysis occurred in 16.09% of patients with bronchiectasis. In a previous survey, the prevalence of bronchiectasis was higher in elderly patients and women in Taiwan [14]; however, there was no significant difference in the incidence of severe hemoptysis between males and females, or among the different age groups in the present study.

Emerging evidence suggests that diabetic patients frequently report respiratory symptoms [15, 16] and are at an increased risk for several respiratory diseases [17–19]. However, bronchiectasis was not observed. We observed that diabetes was a risk factor for severe hemoptysis in patients with bronchiectasis. Animal studies suggest that diabetes may have a direct effect on the pulmonary vasculature. Pulmonary arteries from diabetic rats have been reported to be less responsive to vasodilatation because of increased endothelial dysfunction [20]. Pulmonary vasculature may be affected by diabetic microvascular and macrovascular injuries [16]. Hyperglycemia can lead to complicated infections in the lungs, such as tuberculosis, fungal, and nontuberculous mycobacterial infections, which can cause massive hemoptysis.

The number of lobes with lesions was found to be an independent predictor of massive hemoptysis in patients with bronchiectasis. We observed that the involvement of two lobes was a significant risk factor. Patients with lesions in the right upper lobe were more likely to develop massive hemoptysis. An interesting finding of our study was that lesions located in the left lower lobe rarely caused hemoptysis. In a long-term follow-up study of bronchial artery embolization for massive hemoptysis, the right bronchial artery was found to be the artery most responsible for bleeding [21]. Another previous study found that the right bronchial artery was responsible for hemoptysis in patients with bronchiectasis [22]. The right lobe is supplied by the right bronchial artery. This might be the reason for the higher risk of right lobe involvement. However, why the right upper lobe was not a risk factor is still unknown. Therefore, further studies should be conducted on this topic. At present, the reasons for the lower incidence of massive hemoptysis in the left lower lobe are unclear. No studies have been conducted on this topic. There are also few reliable reports that could explain why the number of two or three lesion lobes was a risk factor for massive hemoptysis. Future prospective studies will be designed to assess the relationship between the number of lesion lobes and massive hemoptysis.

Non-bronchial systemic arteries such as the subclavian, internal mammary, and intercostal arteries, can be a significant source of massive hemoptysis [23]. It may take a longer time for abnormal vessels to develop in patients with bronchiectasis; therefore, patients with a disease course between one and five years may develop severe hemoptysis. However, this needs to be confirmed by clinical evidence.

The limitations of this study are as follows: first, this was a retrospective study performed at only one hospital with a small number of patients with bronchiectasis, who were from the same region. Additional studies should be conducted in other regions to evaluate the risk factors for massive hemoptysis. Second, nontuberculous mycobacterial (NTM) lung disease and pulmonary arterial hypertention are associated with hemoptysis, but these factors were not included in this study owing to the limited number of cases and the lack of necessary examination techniques, such asnontuberculous mycobacteria test and right heart catheterization. Therefore, a multicenter study should be undertaken in the future to include more patients with different diseases that cannot be diagnosed in our hospital and identify more risk factors Third, the actual blood volume may not be consistent with the volume described in the medical record, because patients may not have indicated the exact volume. In addition, not all laboratory finding, such as values of arterial blood gas analysis, were included as potential risk factors because not all lab exams, etc. had been performed in the bronchiectasis patients; therefore, some risk factors might not have been addressed in this study. More studies on the risk factors for massive hemoptysis in patients with bronchiectasis are warranted.

## Conclusions

In this study, we showed that the presence of diabetes, lesions involving two lobes, and lesions involving three lobes were significant risk factors for massive hemoptysis in patients with bronchiectasis. Patients whose disease course was between 1 and 5 years and whose pulmonary lesions were located in the left lower lobe were less likely to develop massive hemoptysis. Lesions in the right upper lobe had a stronger correlation with massive hemoptysis than lesions in other lobes.

## Data Availability

All data generated or analyzed during this study are included in this published article and its supplementary information files.

## Abbreviations

PTMH: Previous times of massive hemoptysis
COPD: Chronic obstructive pulmonary disease
PLT: Platelet
